# Prions of Ruminants Show Distinct Splenotropisms in an Ovine Transgenic Mouse Model

**DOI:** 10.1371/journal.pone.0010310

**Published:** 2010-04-26

**Authors:** Thierry Baron, Anna Bencsik, Eric Morignat

**Affiliations:** Agence Française de Sécurité Sanitaire des Aliments – Lyon, Unité ATNC, Lyon, France; Ohio State University, United States of America

## Abstract

**Background:**

Transmissible agents involved in prion diseases differ in their capacities to target different regions of the central nervous system and lymphoid tissues, which are also host-dependent.

**Methodology/Principal Findings:**

Protease-resistant prion protein (PrP^res^) was analysed by Western blot in the spleen of transgenic mice (TgOvPrP4) that express the ovine prion protein under the control of the neuron-specific enolase promoter, after infection by intra-cerebral route with a variety of transmissible spongiform encephalopathies (TSEs) from cattle and small ruminants. Splenic PrP^res^ was consistently detected in classical BSE and in most natural scrapie sources, the electrophoretic pattern showing similar features to that of cerebral PrP^res^. However splenic PrP^res^ was not detected in L-type BSE and TME-in-cattle, or in the CH1641 experimental scrapie isolate, indicating that some TSE strains showed reduced splenotropism in the ovine transgenic mice. In contrast with CH1641, PrP^res^ was also consistently detected in the spleen of mice infected with six natural “CH1641-like” scrapie isolates, but then showed clearly different molecular features from those identified in the brains (unglycosylated PrP^res^ at ∼18 kDa with removal of the 12B2 epitope) of ovine transgenic mice or of sheep. These features included different cleavage of the main PrP^res^ cleavage product (unglycosylated PrP^res^ at ∼19 kDa with preservation of the 12B2 epitope) and absence of the additional C-terminally cleaved PrP^res^ product (unglycosylated form at ∼14 kDa) that was detected in the brain.

**Conclusion/Significance:**

Studies in a transgenic mouse model expressing the sheep prion protein revealed different capacities of ruminant prions to propagate in the spleen. They showed unexpected features in “CH1641-like” ovine scrapie suggesting that such isolates contain mixed conformers with distinct capacities to propagate in the brain or lymphoid tissues of these mice.

## Introduction

Transmissible spongiform encephalopathies (TSE) such as scrapie in sheep and goats, bovine spongiform encephalopathy (BSE) in cattle and Creutzfeldt-Jakob disease (CJD) in humans, are associated with the accumulation of an abnormal, host-encoded, prion protein (PrP) in infected tissues [1]. Whereas the normal protein (PrPC) is fully sensitive to proteases, the disease-associated prion protein (PrP^d^) is only partly degraded (PrP^res^) by removal of its amino-terminal end. In some of these diseases, such as scrapie, and after transmission in experimental rodent models, PrP^d^ can be detected in the lymphoid tissues that are involved in propagation of the infectious agent.

To some extent, Western blot studies of PrP^res^ in TSE-affected animals reveal distinct molecular features, specifically associated with the “strain” of infectious agent involved in the disease [2]. A typical molecular signature of the BSE agent has notably been identified by Western blot or immunohistochemical analyses of PrP^d^ and such methods can be used to identify the possible presence of BSE in sheep or goats [3], [4]. However a few TSE isolates, showing partial similarities with experimental ovine BSE, have been described in sheep, with a lower molecular mass of unglycosylated PrP^res^ (∼18 kDa) in the brain than in most scrapie cases (∼19 kDa), as also found in ovine BSE. This was first demonstrated in the CH1641 experimental scrapie isolate [5], and then in a few natural scrapie cases in Great Britain and France [6], [7]. Studies of such “CH1641-like” natural isolates in sheep are crucial, especially in view of the recent hypothesis that the BSE epidemic in cattle might have originated from the recycling of an endogenous bovine prion in an intermediate host, such as sheep [8], [9]. Indeed, novel bovine prions, defined by distinct PrP^res^ molecular features with either a higher or lower molecular mass of PrP^res^, in H-type and L-type BSE respectively, than in the classical food-borne BSE, have recently been identified [10], [11]. These unusual cases of BSE putatively represent sporadic cases, like most cases of CJD in humans [12].

Most biochemical studies of TSEs so far have relied solely on the characterization of a main PrP^res^ fragment, which has a gel mobility of ∼18–21 kDa in its unglycosylated form. However, it has recently been shown, using C-terminal antibodies in Western blot studies, that prion discrimination could be facilitated by identifying additional C-terminal PrP^res^ fragments. In human CJD, a ∼13 kDa C-terminal fragment distinguishes type 1 CJD from most type 2 cases, that also differ in the apparent molecular masses of the main PrP^res^ form (∼21 or 19 kDa respectively) [13]. In ruminant TSEs, high levels of an additional ∼14 kDa (unglycosylated form) C-terminal PrP^res^ fragment have been identified in H-BSE in cattle [14] and in “CH1641-like” isolates in sheep [15]. This C-terminal PrP^res^ fragment has not been detected in L-type and classical BSEs.

Bioassays, performed in wild-type mice to identify prion strains from TSE isolates, were reported to identify the biological signature of the BSE agent (incubation periods of the disease, distribution and features of PrP^d^ deposits), but the CH1641 source unfortunately failed to transmit the disease to such mice [5], [16]. Both CH1641 and “CH1641-like” natural isolates were however transmitted in an ovine transgenic mouse model (TgOvPrP4), showing similar PrP^res^ molecular features (i.e. a low apparent molecular of unglycosyslated PrP^res^ (∼18 kDa)) in the brain of both transgenic mice and sheep [17], [18]. Regarding the unusual forms of bovine prions, L-type BSE was successfully transmitted to this ovine transgenic mouse model, whereas H-type BSE failed to transmit [19], and the reverse results were obtained in C57Bl/6 mice [9], [11]. Overall, molecular analyses of PrP^res^ from the mice brains showed that the differences in molecular mass of the main PrP^res^ product, as in the natural host of the disease, were faithfully maintained, together with the identification and proportions of the C-terminal PrP^res^ fragment, if present [14]. The latter allowed the H-type to be clearly distinguished from classical BSE in C57Bl/6 mice [14] and “CH1641-like” scrapie (or CH1641) from both classical and L-type BSEs in ovine transgenic mice [15].

To further our understanding of ruminant TSEs and of their possible relationship, we therefore investigated the detection and biochemical features of PrP^res^ in the spleen of TgOvPrP4 mice compared to those previously described in the mouse brain.

## Results

### PrP^res^ is identified in the spleen of ovine transgenic mice infected with most ruminant TSE sources

Western blot analyses have revealed PrP^res^ in the spleen of TgOvPrP4 ovine transgenic mice infected with most ruminant TSE sources (Table 1). These results were obtained at first passage, after intra-cerebral inoculation of 10% brain homogenates from TSE-infected cattle, sheep or goat.

**Table 1:** Detection and phenotypic features of PrP^res^ in the spleen of TgOvPrP4 mice infected with TSE sources from cattle and small ruminants.

TSE sources	Nb PrPres positive mice in the spleen	Molecular phenotype (1)	
		(+/−)(2)	
		Spleen	Brain
**BSE in small ruminants**			
Ovine BSE (ARQ/ARQ)(3)	3/4	l (−)	l
Ovine BSE (ARR/ARR)(3)	3/3	l (−)	l
Goat BSE	3/3	l (−)	l
**TSEs in cattle**			
C-type	5/5	l (−)	l
L-type	0/5	-	l
TME	0/6	-	l
**Non «CH1641-like» scrapie**			
SSBP/1	4/5	h (−)	h
Natural classical scrapie			
O69	4/5	h (−)	h
O111	4/4	h (−)	h
O171	5/6	h (−)	h
**“CH1641-like” scrapie**			
CH1641	0/7	-	l
Natural isolates			
O104	5/5	h (+)	l and/or h
TR316211	5/5	h (+)	l
06-825	4/4	h (+)	l
06-017	5/5	h (+)	l
06-287	4/4	h (+)	l
06-412	4/4	h (+)	l

(1) h and l refers to the ∼19 kDa or ∼18 kDa molecular masses of PrP^res^, when detectable.

(2) higher (+) or lower (−) molecular mass of PrP^res^ in spleen compared to brain PrP^res^.

(3) amino-acids encoded at codons 136, 154 and 171 of the *prnp* gene.

**Figure 1:**
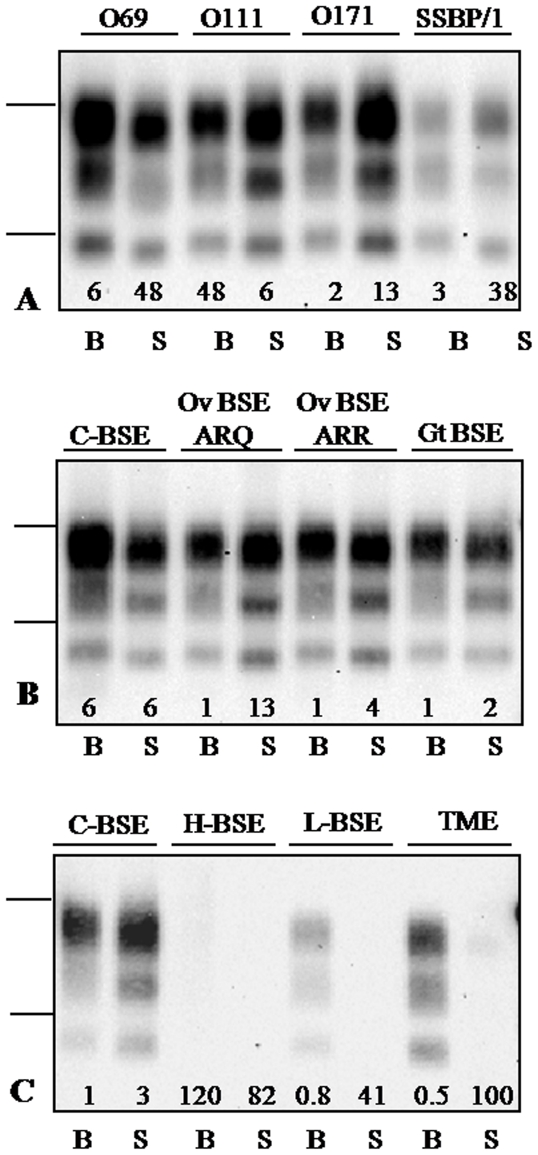
Western blot analysis of PrP^res^ in the spleen and brain of TgOvPrP4 mice. PrP^res^ from the spleen (lanes S) and brain (lanes B) was extracted from TgOvPrP4 mice infected with classical scrapie or bovine TSEs. PrP^res^ was detected using Sha31 (panels B and C) or 12B2 (panel A) antibody. Note that the equivalent tissue quantities loaded per lane, indicated by figures at the bottom of each panel (in tenths of mg), were much higher from the spleens than from the brain in L-type BSE (50×) and TME-in-cattle (200×). Bars to the left indicate the 29.0 and 20.1 kDa marker positions.

The spleens of most ovine transgenic mice (17/20) infected with ovine scrapie, including the SSBP/1 experimental isolate and three natural classical scrapie isolates, were PrP^res^ positive. These four isolates contain PrP^res^ of high apparent molecular mass (∼19 kDa) in the brain, in sheep and after transmission in TgOvPrP4 mice. Analyses of the spleen of these mice, showed a slightly lower apparent molecular mass than in brain, similarly with both 12B2 (Figure 1A) and Sha31 (Figure S2) monoclonal antibodies. Statistical analyses confirmed that the molecular masses of the bi-, mono- and un-glycosylated bands were significantly lower for the spleen than for the brain. Estimates of the mean differences of molecular masses using Sha31 antibody were −0.66 kDa (p<0.0001), −0.79 kDa (p<0.0001) and −0.36 kDa (p<0.0001) for the bi-, mono- and un-glycosylated bands respectively. However, strong 12B2 labeling showed that, as for brain PrP^res^, the 12B2 epitope had not been removed following proteinase K digestion (Figure 1A). Examination on a same gel of the individual spleen samples showed a similar PrP^res^ profile in all the scrapie-infected mice of the same experimental group.

The spleens of 14/15 mice infected with classical BSE were PrP^res^ positive whether they had been infected from cattle, from a goat naturally infected with the BSE agent, or from sheep (homozygous A_136_R_154_Q_171_ and A_136_R_154_R_171_ genotypes) experimentally infected with classical BSE (Figure 1B). The apparent molecular mass of the unglycosylated splenic PrP^res^ was again similar in all the mice examined and lower (0.25–0.4 kDa difference) than that of brain PrP^res^, which had a low apparent molecular mass (∼18 kDa), and was only faintly labeled by 12B2 antibody (data not shown).

### Some TSE strains show a reduced splenotropism in ovine transgenic mice

**Figure 2:**
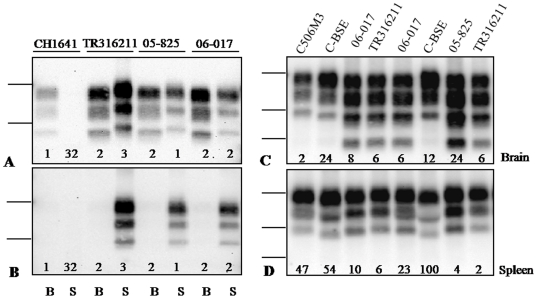
Western blot analysis of “CH1641-like” scrapie isolates in TgOvPrP4 mice. PrP^res^ in the spleen (lanes S in panels A and B, panel D) and brain (lanes B in panels A and B, panel C) were compared in TgOvPrP4 mice infected with natural “CH1641-like” and experimental CH1641 scrapie isolates. PrP^res^ was detected using Sha31 (panel A), 12B2 (panel B) or SAF84 (panels C and D) antibody. Note that the equivalent tissue quantities loaded per lane, indicated by figures at the bottom of each panel (in tenths of mg), were much higher from the spleen than from the brain in CH1641 (30×). The arrow indicates the position of the monoglycosylated band of the C-terminal PrP^res^ product and the head arrows indicate the 22–25 kDa band. Bars to the left indicate the 29.0 and 20.1 kDa marker positions in panels A and B, as well as the 14.3 kDa marker position in panels C and D.

None of the mice inoculated with L-type BSE and TME-in-cattle or with the CH1641 experimental scrapie isolate were PrP^res^ positive in the spleen (Table 1, Figure 1C and 2A). However, all mice inoculated with these three TSE sources were PrP^res^ positive in the brain. Mice inoculated with H-type BSE were negative in both brain and spleen.

#### Splenic PrP^res^ shows major differences with brain PrP^res^ in “CH1641-like” natural scrapie isolates

We then studied six “CH1641-like” natural isolates, characterized by a low apparent molecular mass in the brain of sheep (∼18 kDa), similar to that found in experimental ovine BSE or CH1641. We found that, in contrast with CH1641, all mice infected with the six “CH1641-like” natural scrapie isolates (27/27), were strongly PrP^res^ positive in the spleen by Western blot (Table 1). The presence of PrP^d^ was also confirmed by immunohistochemistry using SAF84 antibody (Figure S1). However, the splenic PrP^res^ in all these mice clearly showed a ∼19 kDa molecular mass (Figure 2A) associated with strong 12B2 labeling (Figure 2B), as previously described in all sources of scrapie with a ∼19 kDa PrP^res^ in brain. This pattern clearly differed from that found in the brains of all mice infected with the five A_136_ R_154_ Q_171_ homozygous sheep, which showed a ∼18 kDa PrP^res^ [15]. For the single case identified in a V_136_ R_154_ Q_171_ homozygous sheep (O104), three of the five mice analyzed showed a predominant ∼18 kDa pattern in the brain, one a predominant ∼19 kDa PrP^res^ pattern (mouse n°5 in Figure S2) whereas the fifth exhibited a mixed pattern [15], [17]. Statistical analyses confirmed that the molecular masses of the bi-, mono- and un-glycosylated bands were significantly higher for the spleen than for the brain (Figure S2). Estimates of the mean differences of molecular masses using Sha31 antibody were respectively +0.32 kDa (p = 0.012), +0.45 kDa (p = 0.014) and +0.65 kDa (p = 0.0005) for the bi-, mono- and un-glycosylated band.

In addition, analyses of splenic PrP^res^ with SAF84 antibody not only confirmed its ∼19 kDa molecular mass (Figure 2D), but also showed that the C-terminal PrP^res^ fragments remained undetectable in the spleen (Figure 2C). This was evidenced by the absence of the ∼14 kDa unglycosylated band and the ∼18 kDa monoglycosylated band (see arrow in Figure 2C) and the lower proportions of the 22–25 kDa band (compare bands indicated by head arrows in Figure 2C and 2D). This latter band results from overlapping of the diglycosylated C-terminal PrP^res^ product, when present, and the monoglycosylated PrP^res^ derived from the main PrP^res^ product. These C-terminal fragments were specifically detected in association with the ∼18 kDa PrP^res^ in the mouse brains from “CH1641-like” natural isolates [15].

## Discussion

This investigation of the possible detection of PrP^res^ by Western blot in the spleens of TgOvPrP4 ovine transgenic mice infected with an extended panel of ruminant TSE sources, confirms that PrP^res^ frequently accumulates in the spleen of TgOvPrP4 mice infected by intra-cerebral route and extends our earlier observations [20]. The apparent molecular mass of splenic PrP^res^ was slightly lower than that accumulated in the brain, with both BSE and scrapie, as previously reported in scrapie-infected sheep [21] or in humans with variant CJD [22]. This presence of PrP^res^ was an unexpected finding since RT-PCR and protein expression analyses by Western blot had failed to detect expression of the *prnp* gene in non nervous tissues in TgOvPrP4 mice during analyses of the entire spleen [20], [23]. PrPC expression was also undetectable in the lymphoid tissues of uninfected mice in a similar transgenic mouse model expressing hamster PrP under the control of the neuron-specific enolase (NSE) promoter. Western blot analysis of the spleen of mice intra-peritoneally infected with the 263K scrapie strain had also revealed low levels of a band consistent with PrP^res^, with a lower apparent molecular mass than in the brain [24]. As PrPC was detected in the peripheral nerves in both hamster and ovine transgenic mouse models, it was hypothesized that PrP^d^ accumulation in the spleen could result from anterograde transport from the brain to the spleen [20], [24]. However, in TgOvPrP4 mice, PrP^d^ accumulation in the spleen was identified by immunohistochemistry in germinal centers in dense areas, reminiscent of follicular dendritic cell clusters [20], as confirmed in the “CH1641-like” natural scrapie isolates in this study (Figure S1). Expression of low levels of PrPC in the spleen, in particular cell types such as the follicular dendritic cells, cannot be fully excluded in either model, and could have remained undetectable in analyses of the entire spleen. The hypothesis of prion replication in particular cell types expressing PrPC, if these are present, remains a possibility. Interestingly, a recent study however showed that the degree of PrP^d^ accumulation in the spleen of experimentally infected sheep did not depend on the PrPC expression level [25]. The major sites of PrP^d^ accumulation were the lymphoid follicles, in which PrP mRNA levels were low, suggesting that PrP^d^ may originate from another site.

Transmission studies then revealed that some TSE sources, unlike classical BSE and most scrapie sources, exhibited reduced splenotropism in TgOvPrP4 mice. Importantly, our study was nevertheless limited to the detection of PrP^res^ by Western blot in the spleen of transgenic mice, and further studies would be required to evaluate the infectivity of the spleen, also in TSE sources that did not show detectable PrP^res^. Several parameters related to the experimental conditions used in our study might have influenced these observations. Firstly, our results could be affected by the species barriers and passage history of the inocula. However, all the TSE sources were examined at a first passage in TgOvPrP4 mice, from cattle, sheep or goat, to limit these possible effects. In the case of the bovine TSE sources, we cannot exclude the possibility that the reduced splenotropism of BSE-L or TME-in-cattle was, at least in part, the result of a species barrier phenomenon, although our data clearly indicate clear differences with classical BSE examined in these same conditions.

The failure to detect PrP^res^ with some TSE sources might be affected by the infectivity levels of the inocula. For example, in wild-type mice, PrP^res^ was detected in the spleen of IM/Dk mice (sinc p7) inoculated with 10% brain homogenates of the 87V strain, but not in VM/Dk mice (also sinc p7) inoculated with 1% brain homogenates, even if terminally infected [26]. The infectious titers of the inocula used in our study have not been determined in TgOvPrP4 mice, but the sources with undetectable splenotropism (BSE-L, TME-in-cattle and CH1641) showed comparable or higher levels of PrP^res^ than the other sources in each experimental group (bovine and ovine isolates), as assessed by ELISA measures of PrP^res^ (data not shown).

All the experiments were also performed solely by intra-cerebral route, this being the most efficient way of experimentally transmitting a prion disease. However, the spleen is known to be readily infected after intra-cerebral inoculation as much of the inoculum escapes from the central nervous system [26], [27]. As we showed, unexpectedly, that TgOvPrP4 mice could also be infected by intra-peritoneal route [20], it would be interesting to analyze the possible transmissibility of these TSE sources and the location of PrP^res^ accumulation after challenge by peripheral routes. Beside the route of inoculation, our study involved the use of transgenic mice, which largely differ from the natural hosts of prion diseases, such as sheep, by the random insertion of transgene copies under the control of non prion regulatory sequences and in a murine genetic background.

The distribution of the infectious agent and/or PrP^d^ is strongly influenced by both the TSE strain and the host of the disease [26], [28]. The identification of distinct lymphotropisms of ruminants TSEs, that is shown in this study in ovine transgenic mice, is not unprecedented and was also demonstrated by experimental transmissions in ruminants. The pathogenic behaviour of classical BSE is strongly influenced by the host, and showed much larger lymphotropism in homozygous A_136_ R_154_ Q_171_ sheep than in cattle [29], [30]. In contrast to classical BSE, recent findings in homozygous A_136_ R_154_ Q_171_ sheep experimentally infected with L-type BSE by intra-cerebral route did not show any evidence of prion accumulation in lymphoid tissues [31]. With regard to TME, whereas reduced extraneural invasion has been shown to be a hallmark of TME infection in mink [32], the TME agent also remained undetectable in the spleen of goats experimentally infected by intra-cerebral route [33]. Finally, transmission experiments with ovine scrapie sources showed that, in contrast to classical BSE or most classical scrapie sources, CH1641 did not readily replicate in the lymphoid tissues after intra-cerebral inoculation [34] and attempts to infect sheep with CH1641 by peripheral routes were also unsuccessful [16].

Our results also demonstrate that PrP^res^, with distinctly different features from the PrP^res^ observed in the brain, could be identified in the spleen of mice infected with “CH1641-like” natural isolates, whereas it could not be detected in the spleen of CH1641-infected mice. These differences are apparent in the spleen, not only as a different N-terminal cleavage of the main PrP^res^ product which migrates at ∼19 kDa and does not remove the 12B2 epitope, but also by the absence of a detectable C-terminally cleaved PrP^res^ fragment, comparable to that previously described in the brains of these mice (∼19 kDa PrP^res^, 12B2 labelled and presence of C-terminal PrP^res^ product) [15]. These differences are unlikely to result from the specific processing in different tissues, since they are only, but consistently, observed in “CH1641-like” isolates and strikingly differ from that found in all other TSE sources. This seems to reflect the presence in “CH1641-like” scrapie of mixed conformers with different abilities to propagate in the spleen and brain or a distinct conformation of PrP^d^ between spleen and brain in these particular isolates. Possible coexistence of both ∼19 kDa and ∼18 kDa PrP^res^ had already been suggested when variable proportions of these two PrP^res^ molecular types were observed in the brains of TgOvPrP4 mice infected with two “CH1641-like” isolates (O100, O104) identified in V_136_R_154_Q_171_ homozygous sheep [6], [17]. In these two sheep, and in contrast with classical BSE, PrP^d^ was clearly identified by immunohistochemical labeling using P4 antibody (93–99 PrP region), not only in some of the brain stem nuclei, such as the dorsal nucleus vagus (X), but also in the lymphoid tissues [6]. The coexistence of ∼18 kDa and ∼19 kDa PrP^res^ has also recently been described in a British sheep, each PrP^res^ phenotype originating from a different brain area [7] and similar results were obtained in a cow that had been intra-cerebrally infected with a pool of British ovine scrapie cases [35]. Our data thus most probably reflect the coexistence of two PrP^res^ molecular types in these “CH1641-like” natural scrapie isolates, a situation which brings to mind the possible co-occurrence of type 1 and type 2 PrP^res^ in some human CJD patients [36]. Whereas the ∼19 kDa PrP^res^ readily accumulates in the mouse spleen, as shown here for all scrapie sources that showed a ∼19 kDa PrP^res^ in the brain of sheep, our data clearly indicate that the ∼18 kDa PrP^res^ from “CH1641-like” natural isolates does not accumulate in the spleen in this mouse model. These results are consistent with those observed in TgOvPrP4 with the CH1641 experimental isolate that failed to propagate in the spleen, and also with those previously reported in the lymphoid tissues of CH1641 experimentally infected sheep [4]. This distinct splenotropism of prions derived from a single TSE source ressembles the situation in TME, in which the DY strain isolated in hamster, associated with a ∼18 kDa PrP^res^, failed to replicate in the lymphoid tissues, in contrast to the HY strain associated with a ∼21 kDa PrP^res^ [37]. More recently, a study of four human vCJD cases inoculated into human transgenic mice, showed that one case produced an alternate PrP^res^ pattern in half of the mice, like that of sporadic CJD (sCJD), whereas all the mice exhibited the typical vCJD molecular pattern in their spleen [38]. Despite this PrP^res^ pattern of sporadic CJD in the brain, only the PrP^res^ pattern of vCJD was again detected in the spleen after the second passage in such mice, suggesting that the vCJD agent was still propagated as a minor component in the brains, but was preferentially replicated in the spleen. Overall, this example in a human disease shows that distinct propagation of prions in cerebral and lymphoid tissues of the same TSE-infected animal is not unprecedented.

Finally, although the precise cellular origin of splenic PrP^d^ in TgOvPrP4 ovine transgenic mice still needs to be determined, our data suggest that this ovine transgenic mouse model properly reflects the behavior of the different TSE agents in sheep with regard to their capacity to propagate in the spleen. The unexpected behavior of natural “CH1641-like” ovine TSE isolates, in which prions distinct from those found in the brain accumulated in the spleen of TgOvPrP4 mice, suggests the possibility of a mixture of two strains. The TSE agents associated with the two PrP^res^ phenotypes identified in the brain or spleen still need to be biologically characterized and, more generally speaking, the basic mechanisms underlying the variable tissue or cell tropisms of different prions inside the central nervous system and in the peripheral tissues are still little understood.

## Materials and Methods

### Ethics statement

Experiments were performed in the Biohazard prevention area (A3) of the author's institution with the approval of the Rhône-Alpes Ethical Committee for Animal Experiments and following the guidelines of the French Ethical Committee (decree 87-848) and European Community Directive 86/609/EEC.

### Mouse transmission studies

Experimental transmissions of a variety of natural or experimentally-produced TSE sources from small ruminants and cattle (Table 1) were previously reported, after intra-cerebral inoculation in a transgenic mouse model (TgOvPrP4) overexpressing the ovine PrP protein (A_136_R_154_Q_171_ allele) under the control of the neuron-specific enolase (NSE) promoter [23]. The TSE sources from small ruminants included (i) natural or experimental scrapie, including the SSBP/1 and CH1641 experimental scrapie isolates [18], [23], (ii) “CH1641-like” natural scrapie, characterized by a similar apparent molecular mass of PrP^res^ to that of ovine BSE or CH1641 experimental scrapie isolate (∼18 kDa) [15], [17], [39], and (iii) BSE [18], [40], [41]. All the “CH1641-like” natural isolates were identified in A_136_ R_154_ Q_171_ homozygous sheep, except the O104 isolate which was from a V_136_ R_154_ Q_171_ homozygous sheep [6], [39]. From cattle, the food-borne classical BSE, the H-type and L-type atypical forms of BSE, as defined by their molecular differences with classical BSE, and transmissible mink encephalopathy (TME) experimentally transmitted to cattle were included [15], [19]. Only H-type BSE consistently failed to transmit the disease to TgOvPrP4 mice, as assessed by the absence of PrP^res^ in the brain [19].

### Biochemical studies of PrP^res^

PrP^res^ was extracted and detected as previously described [15], from mice infected at first passage performed by inoculation of 10% brain homogenates with the different TSE sources from ruminants. PrP^res^ was extracted from the entire spleens by treating the spleen homogenates with collagenase (100 µg/100 mg spleen in a 1 ml total volume) and DNAse (64 µg/100 mg spleen in a 1 ml total volume) for 1h at 37°C, then with proteinase K (24 µg/100 mg spleen in a 1.2 ml volume) for 1h at 37°C. After 15% SDS-PAGE and electro-blotting on nitrocellulose membranes, PrP^res^ was detected with 12B2 (340 ng/ml) (kindly provided by Dr J. Langeveld, CIDC-Lelystad, The Netherlands), Sha31 (1/10 from TeSeE Bio-Rad sheep and goats kit)(Bio-Rad, France) or horseradish peroxidase-labeled SAF84 (500 ng/ml)(SPI-Bio, France), against the 93-WGQGG-97, 148-YEDRYYRE-155 and 167-RPVDQY-172 ovine PrP sequences respectively. These antibodies allow detailed epitope mapping of the protease-resistant prion protein accumulating in the infected tissues, and molecular discrimination between different strains [7], [15], [34]. Peroxidase-labelled conjugate anti-mouse IgG (H+L)(1/2500 in PBST)(ref 1010-05)(Clinisciences, France) was used to detect 12B2 and Sha31 antibodies. Streptavidin (5 ng/ml)(S5512) was added to the conjugate solution. Bound antibodies were then detected by direct capture with the Versa Doc (Biorad) analysis system, using the ECL chemiluminescent substrate (Amersham, France). Quantitative studies were performed using Quantity One (Biorad) software and the apparent molecular masses were measured by comparing the positions of the PrP^res^ bands with a biotinylated marker (B2787)(Sigma, France).

The profiles of PrP^res^ extracted from the individual spleens (17 and 27 mice respectively for “non CH1641-like” and “CH1641-like” ovine scrapie, 14 mice for classical BSE) were first compared by loading the samples of each mouse belonging to the same experimental group on the same gel, as performed for the brains of these mice in previously published studies. The PrP^res^ profiles from brain and spleen were then compared by performing Western blot analyses of the brain and spleen in a panel of individual mice loaded lane by lane on the gel (see Figure 1). This allowed accurate determination of the differences in molecular masses of the PrP^res^ bands between brain and spleen from the same animal as indicated by “+” (higher in the spleen) or “−” (lower in the spleen) in Table 1.

### Statistical analyses

For “CH1641-like” and “non CH1641-like” ovine isolates, 17 and 46 measures of the molecular masses were made respectively for the di-, mono- and unglycosylated bands, on the spleen and brain of 9 and 13 mice respectively, with one to four replications per mouse. Figure S2 shows the differences of molecular masses between spleen and brain for the three PrP^res^ glycoforms from the 22 mice infected with the two categories of isolates.

Six linear mixed effects models (one model for each combination isolate/band) were adjusted to estimate the difference of molecular mass between spleen and brain. A model was written as follows :
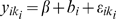
with 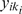 the k_i_^th^ replication of the difference of molecular mass between spleen and brain of the mouse i, 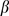 the mean effect of the difference of molecular mass and 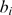 a random effect for the mouse effect with 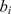
∼N(0,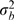) and 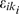
∼N(0,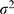). The parameter 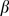 was of particular interest as it gives an estimation of the difference of molecular masses between spleen and brain and the p-value of the t-test associated with 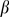 was used to assess if it was significantly different from zero. Assessment of the models was done by graphical examination of the residuals.

Statistical analysis was performed with the package nlme (Jose Pinheiro, Douglas Bates, Saikat DebRoy, Deepayan Sarkar and the R Core team (2009). nlme: Linear and Nonlinear Mixed Effects Models. R package version 3.1–96) of the R software (R Development Core Team (2009). R: A language and environment for statistical computing. R Foundation for Statistical Computing, Vienna, Austria. ISBN 3-900051-07-0, URL http://www.R-project.org).

## Supporting Information

Figure S1 PrP^d^ immunohistochemical analysis of TgOvPrP4 mouse spleen. Mice were infected with the SSBP/1 (left panel) or 05-825 natural scrapie source (right panel), using SAF84 monoclonal antibody [20], [42]. PrP^d^ is revealed by the presence of black deposits of DAB intensified by using chloride nickel within the follicles. Both the location and shape of the positively labeled-cells are totally similar to those described in previous studies in this model [20] and are probably follicular dendritic cells.
(1.58 MB TIF)

Figure S2 Differences of molecular masses of the PrP^res^ glycoforms between the spleen and brain of individual mice infected by “CH1641-like” or “non CH1641-like” ovine scrapie isolates. PrP^res^ extracted from brain and spleen of individual mice (Mouse) were loaded lane by lane and differences of the three PrP^res^ glycoforms were measured by repeated (1–4×)(Rep) Western blot analysis.
(10.50 MB TIF)
