# Supplementary figures and images for: Prions of Ruminants Show Distinct Splenotropisms in an Ovine Transgenic Mouse Model
Source: PLoS One. 2010 Apr 26;5(4):e10310. doi: 10.1371/journal.pone.0010310 (PMC2859945; doi:10.1371/journal.pone.0010310)

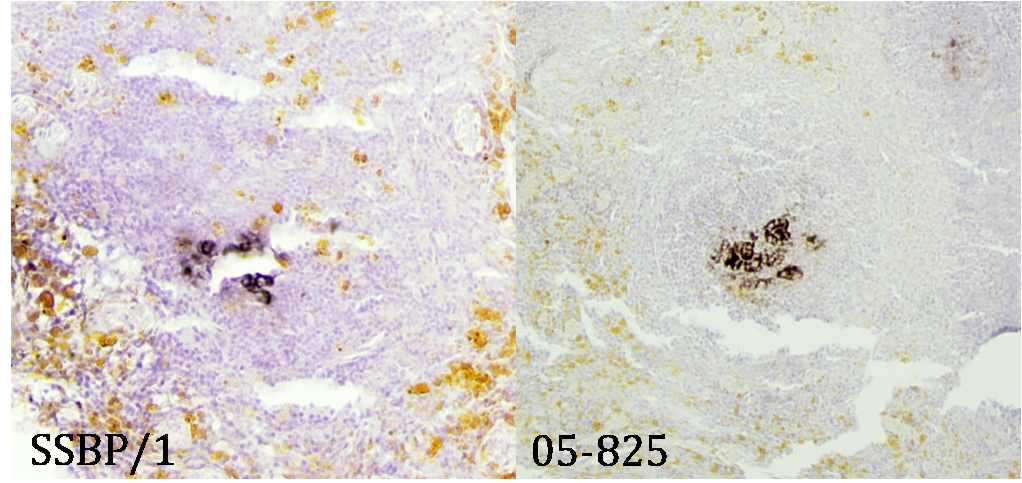

Supplement: Figure S1 — PrPd immunohistochemical analysis of TgOvPrP4 mouse spleen. Mice were infected with the SSBP/1 (left panel) or 05-825 natural scrapie source (right panel), using SAF84 monoclonal antibody [20], [42]. PrPd is revealed by the presence of black deposits of DAB intensified by using chloride nickel within the follicles. Both the location and shape of the positively labeled-cells are totally similar to those described in previous studies in this model [20] and are probably follicular dendritic cells. (1.58 MB TIF) [file pone.0010310.s001.tif]

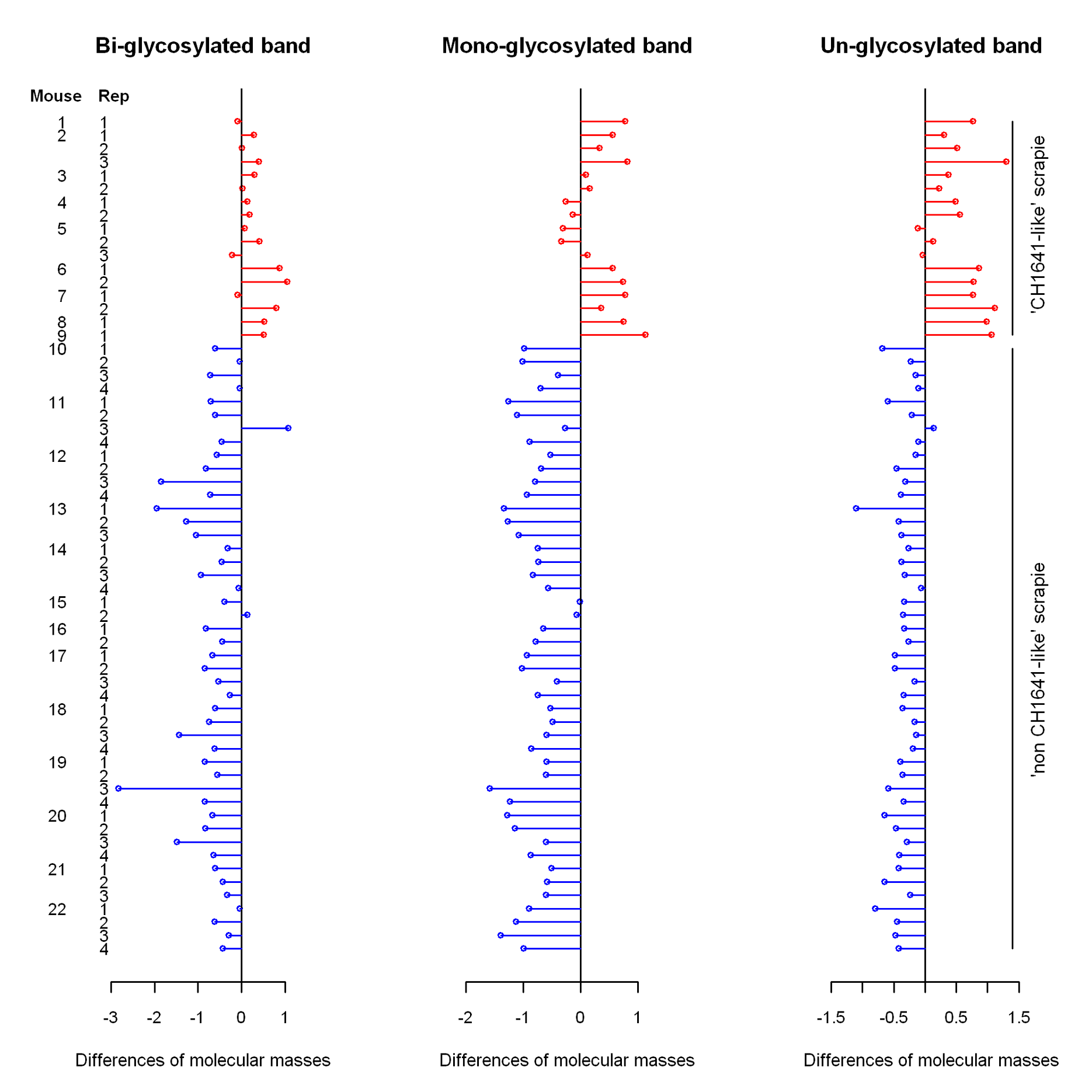

Supplement: Figure S2 — Differences of molecular masses of the PrPres glycoforms between the spleen and brain of individual mice infected by “CH1641-like” or “non CH1641-like” ovine scrapie isolates. PrPres extracted from brain and spleen of individual mice (Mouse) were loaded lane by lane and differences of the three PrPres glycoforms were measured by repeated (1–4×)(Rep) Western blot analysis. (10.50 MB TIF) [file pone.0010310.s002.tif]
